# Intravascular large B-cell lymphoma involving gastrointestinal stromal tumor: a case report and literature review

**DOI:** 10.1186/s13000-015-0446-2

**Published:** 2015-12-16

**Authors:** Fen Zhang, Xinlan Luo, Yu Chen, Yanhui Liu

**Affiliations:** Department of Pathology, Guangdong General Hospital, Guangdong, Academy of Medical Science, Guangzhou, 510080 China

**Keywords:** Intravascular large B-cell lymphoma, Gastrointestinal stromal tumor, Synchronous

## Abstract

**Background:**

Intravascular large B-cell lymphoma is a rare and aggressive lymphoma with a dismal prognosis. Synchronous intravascular large B-cell lymphoma involving gastrointestinal stromal tumor has not previously been documented.

**Case Presentation:**

We report a case of a 61-year-old Chinese woman who presented with high fever of unknown origin for 20 days, and hematemesis, melena for 2 days. A computed tomography scan revealed a mass lesion located in the anterior wall of the stomach. Surgery was performed to remove the tumor and histopathology showed a gastrointestinal stromal tumor and a synchronous intravascular large B-cell lymphoma. The patient refused further treatment and died 4 months after surgery.

**Conclusions:**

To the best of our knowledge, this case represents the first report of synchronous intravascular large B-cell lymphoma involving a gastrointestinal stromal tumor.

## Background

According to the actual WHO Classification of Tumors of Haematopoietic and Lymphoid Tissues 2008, intravascular large B-cell lymphoma (IVLBCL) belongs to the category of mature B-cell neoplasms. It is a rare type of extranodal large B-cell lymphoma with selective growth of lymphoma cells within the lumina of small to intermediate calibre vessels. It typically occurs in elderly persons. It may be present in any organ in the absence of lymphadenopathy with various systemic symptoms, such as fever of unknown origin, general fatigue, marked deterioration in performance status, and neurological alteration. The absence of typical clinical manifestations and the aggressive behavior of IVLBCL frequently make accurate and immediate diagnosis difficult. Synchronous IVLBCL and menigioma or breast cancer has been reported [1, 2].

Stomach is one of the most common sites for malignant extranodal lymphomas. A wide variety of histological subtypes have been reported, the most of which are mucosa-associated lymphoid tissue(MALT) lymphoma and diffuse large B cell lymphoma. IVLBCL in stomach has not yet been described in the literature. Gastrointestinal stromal tumor (GIST) is a rare mesenchymal tumor. Stomach is the most common site of involvement of GIST. There are several reports of concomitant gastric GIST and MALT lymphoma in the English literature [3, 4]. To the best of our knowledge, this case represents the first report of synchronous IVLBCL involving a gastric GIST.

## Case presentation

A 61-year-old woman presented with a 20-day course of high fever of unknown origin, general fatigue, and two-day history of hematemesis and melena, was admitted to Guangdong General Hospital in July 2013. Physical examination was normal. Abnormal laboratory values included; hemoglobin: 51 g/L (reference interval 115–155 g/L), sodium concentration: 126 mmol/L(reference interval 136–145 mmol/L), total protein: 59 g/L(reference interval 60–80 g/L), white protein: 14 g/L(reference interval 35–55 g/L), lactate dehydrogenase(LDH): 1233 U/L(reference interval 109–245 U/L). Activated partial thromboplastin time: 58 s(reference interval 30–45 s), plasma fibrinogen(Fg): 5 g/L (reference interval 1.9–4 g/L). Computed tomography(CT) scan of the abdomen showed a heterogeneous mass of 2.5 cm in diameter in the anterior wall of the stomach which was 4 cm away from the cardia. Surgical examination showed no lymphadenopathy, hepatosplenomegaly or other masses in the abdomen. Partial gastrectomy was performed.

**Fig. 1:**
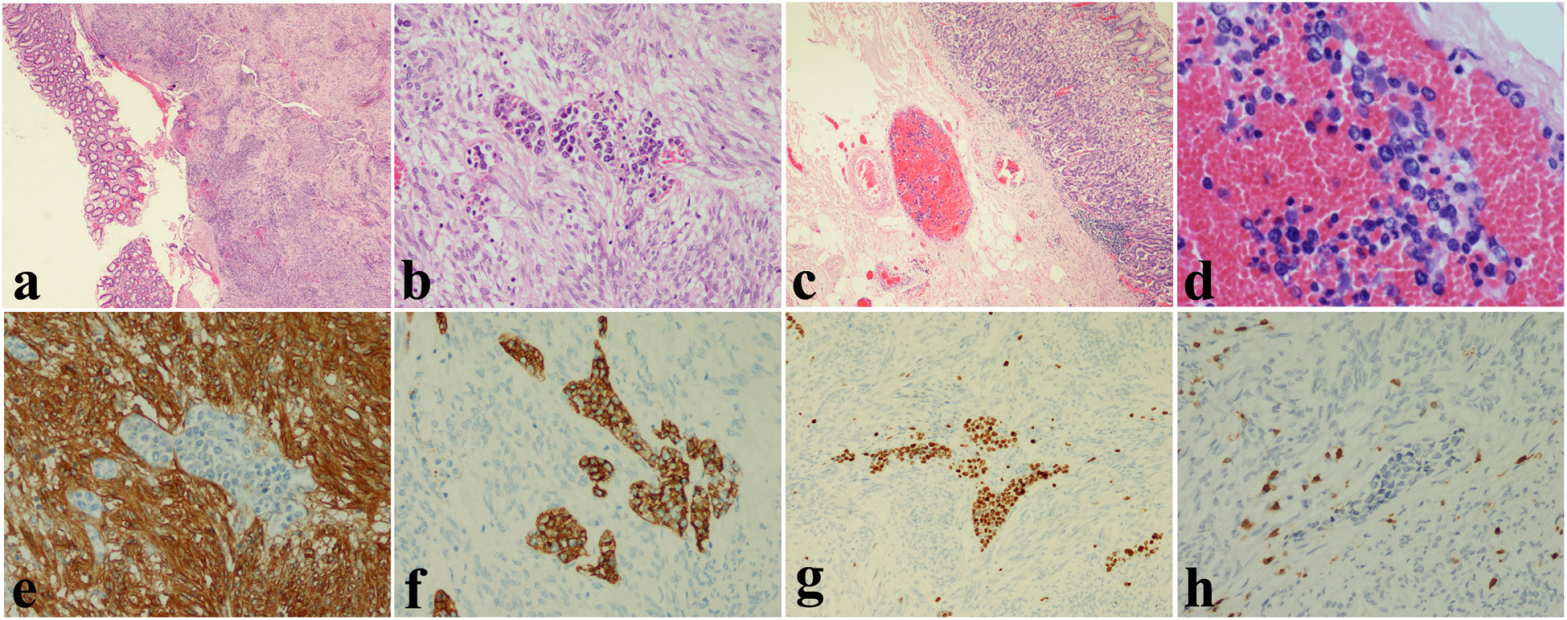
Morphology and representative Immunohistochemical staining of the tumor. The tumor had an ulcer on the surface (**a**, ×20). It was composed of spindle-shaped cells and had atypia lymphoid cells within intratumoural blood vessels (**b**, ×200). There were the same cells in surrounding submucosa blood vessels (**c**, ×40 and **d**, ×400). The spindle cells were CD117 positive (**e**, ×200). The lymphoid cells were CD20 positive (**f**, ×200). The Ki67 proliferation index of the lymphoid cells was close to 100 % (**g**, ×100). The lymphoid cells were CD3 positive (**h**, ×200)

Gross examination revealed the tumor was located in submucosa, and had an ulcer on the surface. Histologically, the tumor was monomorphic, composed of spindle-shaped cells. Mitotic activity ranged between 2 and 4 in 50 high power fields. Immunohistochemical examination showed that it was positive for CD34 and CD117, but negative for smooth-muscle actin. The Ki67 proliferation index was about 2 %. These findings were consistent with a diagnosis of gastrointestinal stromal tumor. Further inspection revealed multifocal, scattered malignant cells which were exclusively within the lumina of intratumoural and surrounding submucosa blood vessels. These cells were discohesive and displayed a lymphoid phenotype with a rounded morphology, hyperchromatic round or irregular nuclei with prominent nucleoli, and a small amount of amphophilic cytoplasm. Immunohistochemical examination showed these cells were CD20, MUM1 positive, and CD10, CD3 negative. The Ki67 proliferation index was close to 100 %. The morphology and immunohistochemical profile indicated a diagnosis of intravascular B-cell lymphoma involving gastrointestinal stromal tumor (Fig. 1, a-h). The patient refused further treatment and died 4 months after the surgery.

## Discussion

Our patient showed a series of nonspecific symptoms as fever of unknown origin, general fatigue, gastrointestinal hemorrhage, severe anemia, hypoalbuminaemia, hyponatremia, elevated LDH level and coagulation disorders. The presumptive diagnosis was GIST. Histopathologically confirmed ulcer on the tumor might explain the symptoms of gastrointestinal hemorrhage and anemia. Previous retrospective analysis of GISTs also concluded that these two symptoms were the most common clinical presentations for a GIST [5]. However GIST alone failed to identify the reason for the other symptoms. Further inspection revealed IVLBCL both in the tumor and gastric submucosa around the tumor, and all the symptoms of the patient could be explained by an IVLBCL.

Intravascular large B-cell lymphoma has two main clinical patterns, a Western pattern characterized by neurological or cutaneous involvement and an Asian variant featuring multiple organ failure, hepatosplenomegaly, pancytopenia and hemophagocytic syndrome. However, an immediate diagnosis of IVLBCL in many patients still remains challenging, because tumor involvement can occur in any organ, even without apparent clinical signs. Hematological abnormalities and increased LDH levels commonly occur [6], but these manifestations are also common in other hematological diseases. Moreover, increased soluble interleukin-2(IL-2) receptor levels have been reported in patients with IVLBCL [6], Furthermore, soluble LR11(sLR11) levels are significantly higher in patients with IVLBCL than in those with other hematological malignancies, and may represent a potentially powerful diagnostic indicator for IVLBCL. In addition, the significant reduction of sLR11 levels upon disease remission suggests that this protein may represent a sensitive marker for disease monitoring [7]. But a widely used specific serum biomarker that is directly derived from IVLBCL cells has not yet been identified.

The organs selected by the physician for biopsy are key to accurate diagnosis. In Asian cohorts, the most relevant diagnostic site seems to be the bone marrow [8]. There are several cases reported as IVLBCL colonizing preexsistent hemangiomas [9–11]. Actually we have a case of IVLBCL involving hepatic cavernous hemangioma(unpublished case). Skin lesions including telangiectasia, senile angioma-like eruption, purpuric plaques and indurated erythematous plaques were present in one third of patients. An early diaganosis could be made by biopsy of these lesions [12–14]. Even in patients without skin lesions, the usefulness of random skin biopsy (RSB) for the diagnosis of IVLBCL has been suggested [15–18]. Asada et al. recommended deep biopsy involving subcutaneous fat because the affected vessels were located in the deep dermis or subcutaneous adipose tissue in cases [18].

In pathological examination, neoplastic cells of IVLBCL are large lymphoid cells with prominent nucleoli and express B-cell-associated antigens like CD19, CD20, CD22 and CD79a, and almost all are CD10 negative cases showing a non-germinal center pattern with IRF4/MUM1 positivity. They are negative for T cell markers like CD3, CD5. Factors underlying the exclusively intravascular location of the tumor cells remain enigmatic but could be related to the absence of certain endothelial adhesion proteins that has been demonstrated in IVLBCL, such as CD29 and CD54 [19].

Clinical outcomes of IVLBCL were extremely dismal before the rituximab era. However the recent development of rituximab-containing chemotherapy has improved the outcome of IVLBCL: a retrospective analysis of Western and Asian cohorts reported 3-year overall survival rates of 81 and 60 % respectively [20, 21]. But our patient refused further treatment because she could not afford the expensive treatment costs, and died 4 months after surgery.

## Conclusion

In conclusion, we describe the first case of synchronous ILBCL and GIST of the stomach, the occurrence of a GIST in this particular case could be considered fortuitous. We want to emphasize that nonspecific clinical findings as fever and progressive deteriorated general conditions in combination with elevated LDH and low albumin levels should should raise the suspicion for lymphoma in general and after exclusion of these even for rare lymphoma like IVLBCL. Absence of lymphoadenomegaly, hepatosplenomegaly, bone marrow involvement, or other solid neoformations does not rule out a lymphoproliferative disorder.

## Consent

The informed consent was obtained from the patient’s relatives for the publication of this case report and any accompanying images. A copy of the written consent is available for review by the Editor-in-Chief of this journal.

## Abbreviations

IVLBCL: intravascular large B-cell lymphoma
MALT: mucosa- associated lymphoid tissue
GIST: gastrointestinal stromal tumor
LDH: lactate dehydrogenase
